# Exploring the Gender Gap in Teleworking from Home. The Roles of Worker’s Characteristics, Occupational Positions and Gender Equality in Europe

**DOI:** 10.1007/s11205-023-03133-6

**Published:** 2023-05-24

**Authors:** Stefanie Kley, Thordis Reimer

**Affiliations:** Department of Social Sciences, Universität Hamburg, Hamburg, Germany

**Keywords:** Gender equity, Telecommute, Gender gap, Occupational gender segregation, Multilevel analyses, Europe

## Abstract

Previous research suggests an under-representation of women among teleworkers before the Covid-19 pandemic. However, we know little about whether such a gender gap was substantial, and whether it could be explained by occupational gender segregation. We explore whether a gender gap in regularly teleworking existed in the EU-28 and analyse its possible constituents, drawing on data from the European Working Conditions Survey 2015. To form a group of potential teleworkers, the analytical sample was restricted to employees who made use of information and communication technology (N ≈ 16,000). Country fixed effects regression and multilevel models were applied. The results show that women were under-represented among teleworkers compared to men, also when occupational gender segregation is taken into account; the remaining gender gap in telework is estimated at 10%. For women, working part-time and working in the private sector was associated with lower incidences of telework, but not for men. Country characteristics explain a small but significant share of telework incidence. In countries that rank high on the Gender Equality Index and have a large public sector, telework was widespread, whereas it was less present in countries with higher shares of women in the fields of science and engineering. The findings support the view that the gender gap in teleworking from home is a matter of historically grown gender inequality.

**Supplementary Information:**

The online version contains supplementary material available at 10.1007/s11205-023-03133-6.

## Introduction

At the onset of the Covid-19 pandemic in spring 2020, the share of full-time telecommuters in the European Union jumped close to 40% of the workforce and continued to rise to 48%. In contrast, in 2019 the share of employees who at least occasionally worked from home was only about 10% (Milasi et al., 2020; Eurofound, 2021). Thus, due to the Covid-19 induced restrictions on movement, many employees who were previously not allowed to work outside their employer’s premises now had this very experience. An important question for employees and employers, as well as for policy-makers in various political fields is therefore, whether and for whom this new practice of working from home will last. In this situation, it is insightful to analyse in more detail which groups of workers were regular teleworkers before the pandemic, as the new situation has put the opportunity to work from home as a possible source of social inequality on the research agenda. Contrary to research on forms of flexible working involving workers’ control over *when* they work (Glass & Estes, 1997; Kelly et al., 2011; Allen et al., 2013), worker’s control over *where* they work is understudied, particularly outside the Anglo-Saxon context (Chung & van der Lippe, 2020).

Previous studies suggest that women were under-represented among teleworkers in Europe before the Covid-19 pandemic (Eurofound, 2007, 2010; Brenke, 2014; López-Igual & Rodríguez-Modroño, 2020), although the findings vary considerably with the type and frequency of telework under study (Bailey & Kurland, 2002; Eurofound and International Labour Office, 2017; López-Igual & Rodríguez-Modroño, 2020), and also with the country context (Eurofound, 2007, 2010; Milasi et al., 2020). Multivariate studies that comprehensively analyse telework opportunities or its use are scarce (exceptions are Peters et al., 2004; Wight & Raley, 2009; Elldér, 2019; López-Igual & Rodríguez-Modroño, 2020; for the U.S., e.g. Singh et al., 2013); however, these studies did not focus on gender. Moreover, the underlying definitions of teleworking vary, which makes it difficult to compare the findings (Mokhtarian, 1991; Sullivan, 2003; Mokhtarian et al., 2005; Allen et al., 2015). What is needed is research that explores teleworking on a regular basis that is carried out in a predictable manner as a normal part of the working week. Such regular telework is what we have in mind when we think about telework as an increasingly widespread form of work. It is not taking work home because at the end of the week there was so much work still left to do; and it is not working from home occasionally because the child is sick. In this paper, we use the definition of telework developed by Eurofound and the ILO that considers both the use of information and communication technology (ICT) and the frequency of working remotely (Eurofound and International Labour Office, 2017). The term ‘telework’ is the most commonly used term to refer to employees’ work performed with the help of ICT from outside the employer’s premises, i.e. ‘mobile’ (labelled “T/ICTM” in the report of Eurofound and International Labour Office 2017). In the course of analyses, the sample will be restricted to employees who work with ICT in order to analyse their probability of regularly teleworking from home (“T-ICTM from home” according to the report of Eurofound and International Labour Office 2017). Especially in the United States, India, and Japan, home-based teleworkers are also referred to as “telecommuters” (Mokhtarian et al., 2005; Eurofound and International Labour Office, 2017). As our focus is on home-based teleworkers, we use the terms “teleworkers” and “telecommuters” interchangeably. An analysis of different types of telework in Europe, i.e. from home and other places, was presented byLópez-Igual and Rodríguez-Modroño (2020).

The focus on ICT work allows us to distinguish telework from other home-based work such as piecework, farming, or homemaking (cf. Sullivan, 2003). Little is known about the question whether a gender gap in regular teleworking is systematic in Western societies, and if so, whether it is mediated by gender-specific distributions of work characteristics. With this paper, we want to contribute to closing this research gap.

Theoretically, our research agenda builds on the understanding of gender inequalities as disadvantages that women experience relative to men who are otherwise their social equals (Saltzman Chafetz, 2006; Dorius & Firebaugh, 2010), e.g. in family background, educational level, and labour market position. The framework of occupational gender segregation (e.g. Blackburn et al., 2002) is used to explore the constituents of the gender gap in telework.

In a *first step*, we investigate in which occupational groups the use of ICT was substantial before the Covid-19 pandemic. The distribution of men and women within occupational groups that are or are not characterized by substantial ICT use, gives an impression about the relevance of *horizontal* gender segregation with regard to telework. In a *second step*, we restrict the analytical sample to ICT users, in order to analyse the constituents of the remaining gender gap in telework within the group of *potential* teleworkers. With this analytical strategy, we minimize the influence of horizontal gender segregation and put the analysis’ emphasis on gender inequality. In a *third step*, we explore the degree to which the gender gap in telework is attributable to country-level particularities. Although it is not possible to explore all possible country-level influences in this paper, it would be incomplete without a glance at selected characteristics that reflect aspects of occupational gender segregation on telework, among them the realized level of “gender equality” (European Institute for Gender Equality (EIGE)) in the countries studied.

The data come from the 2015 European Working Conditions Survey (EWCS) comprising about 28,200 employees in the first step of analysis and an analytical sample of about 16,000 in the second step, living in one of the 28 countries of the European Union (EU). We apply logistic regression with country fixed effects and two-level mixed-effects logistic regression to estimate the odds of teleworking from home dependent on socio-economic and work characteristics, taking the embeddedness of teleworkers in country-specific labour markets and other country particularities into account.

## Occupational Gender Segregation and Telework Among Men and Women

### Placing Telework Within the Framework of Occupational Gender Segregation

Within the framework of occupational gender segregation, a vertical and a horizontal dimension of gender segregation can be distinguished (Blackburn et al., 2000, 2002; Steinmetz, 2012). Vertical gender segregation results in higher shares of men compared to women in advanced occupational positions, with more supervisory competence, better opportunities for career progression and/or higher pay (Bishu & Alkadry, 2017, provide a systematic review of the literature). Horizontal gender segregation results in a concentration of men and women in particular occupations or fields of work (e.g. Hakim 2006; Thébaud & Charles, 2018). Whereas it is consensus that vertical gender segregation bears *gender inequalities* – empirically most often an advantage of men over women – this is not so clear with regard to horizontal segregation. In contemporary Western societies, many work tasks are widely presumed to be intrinsically masculine or feminine (Faulkner, 2000; Nosek et al., 2002; Des Jardins, 2010). As occupational choice might reflect gendered affinities and aspirations, the concentration of men and women in specific fields of work can be seen to reflect *gender difference* (e.g. Blackburn et al., 2000; Saltzman Chafetz, 2006). It is then an empirical question as to what extent aspects of gender difference may or may not be related to gender inequality.

With regard to telework, the “nature” of occupations, i.e. the main tasks to perform, is a central issue, as many occupations do not allow for substantial telework (Sullivan, 2003; Singh et al., 2013; Brenke, 2016), e.g. the work of nursery teachers, florists, geriatric nurses, and car mechanics. With regard to economic sector, telework was most widespread in information technology and other communication services; in knowledge-intensive business services; in education; and in publishing, audio-visual and broadcasting services (Milasi et al., 2020; Elldér, 2019). Professionals who most often teleworked were teachers at schools and universities; office staff; IT staff; managing directors, officials, and ministers (Brenke, 2014). Telework was rarely available in simple and qualified manual activities; in qualified services outside the commercial area, for instance in personal services; and in simple commercial and administrative activities, e.g. for clerks and sales workers (Brenke, 2014; Milasi et al., 2020; Singh et al., 2013). Consequently, telework was positively associated with educational level (Peters et al., 2004; Elldér, 2019; López-Igual & Rodríguez-Modroño, 2020), and professional position (Peters et al., 2004; Brenke, 2016; López-Igual & Rodríguez-Modroño, 2020), but the findings about associations of telework with income were mixed (Singh et al., 2013; Elldér, 2019). Given women’s under-representation in high status positions (Bishu & Alkadry, 2017) and the fields of science, technology, engineering, and mathematics (STEM) (Mann & DiPrete, 2016; Thébaud & Charles, 2018),

*we hypothesize to find fewer women regularly teleworking from home compared to men also among ICT employees (H1).*

### Is Teleworking a Gender Issue?

Implementing more practices that increase workplace flexibility, among them telecommuting, is seen as a way to help working families, particularly working women (Kizza, 2017). There is some evidence that telework can help working parents to juggle work and family life better (Sullivan & Lewis, 2001; Hill et al., 2003; Crosbie & Moore, 2004; Sullivan & Smithson, 2007; Wheatley, 2012a; Powell & Craig, 2015; Reimer, 2015), as it better allows workers to schedule work demands around family obligations. Thus, telework is seen as one type of flexible employment enhancing worker’s control over *where* they work, another is working part-time and flexible hours, enhancing worker’s control over *when* they work (Kelly et al., 2011; Allen et al., 2013). Whereas part-time is a female-dominated flexible work arrangement for combining work and family (Drobnic, 2000; Boeckmann et al., 2015), analyses based on time-diary data suggest that telework is often used by men in this regard (Wight & Raley, 2009; Reimer, 2015; Glass & Noonan, 2016). As part-timers enjoy higher schedule flexibility compared to full-timers, it might be the case that they were less interested in telework. This notion is corroborated by findings of a reduced preference for telework among part-timers (Peters et al., 2004); another study found equal preferences for telework in part-timers and full-timers, but reduced telework options for part-timers (Singh et al., 2013). Yet another finding was that part-timers teleworked less compared to full-timers (López-Igual & Rodríguez-Modroño, 2020). Considering that telework might be seen as a substitute for part-time work, while the latter is the dominant work arrangement for mothers,

*we hypothesize that part-time working was associated with a reduced probability of teleworking (H2a), particularly for women (H2b).*

Telework in the EU seems to have been particularly prevalent in public administration (Eurofound, 2007), although the differences in access to and use of telework options between the public and the private sector varied considerably between countries (Brenke, 2016; cp. Eurofound and International Labour Office 2017). As the extent to which public sector employers offer equal opportunities for women and parents is under public scrutiny, opportunities for telework might be more widely available in the public sector compared to the private sector, and they might be granted to part-timers and full-timers equally.

*We hypothesize that working in the private sector was associated with a reduced probability of teleworking compared to working in the public sector (H3a), particularly for women (H3b).*

The commute between home and the workplace might also be a source of gender differences with regard to telework. One might expect that workers with long commutes are particularly interested in teleworking, as spending time on commutes between home and the workplace is often stressful (Stutzer & Frey, 2008; (Wheatley, 2012b), and often interferes with family life (Huinink & Feldhaus, 2012; Kley & Feldhaus, 2018; Brömmelhaus et al., 2020). For the Netherlands it was found that long commutes of at least one hour in each direction were positively associated with both workers’ preferences for and practice of telecommuting (Peters et al., 2004). Singh (2013) found similar positive associations in the US between employees’ telework options and choices when they had a one-way commuting distance of more than 20 miles. Accordingly, living in rural areas was found to be positively associated with telework (López-Igual & Rodríguez-Modroño, 2020; Singh et al., 2013), whereas employment accessibility was negatively associated (Singh et al., 2013). With regard to possible gender differences, it was found that married and cohabiting women, as well as mothers, were under-represented among long-distance commuters in Europe (Meil, 2010; Feldhaus & Schlegel, 2013), suggesting that women were more inclined to avoid long commutes than men.

*We therefore hypothesize that teleworking from home was associated with avoiding lengthy commutes (H4a), particularly for women (H4b).*

The current state of research suggests further associations with telework that should be considered in order to avoid biased estimates. With regard to socio-demographic characteristics it was found that having children in the household is associated with greater eligibility to (Singh et al., 2013; Elldér, 2019), preference for (Peters et al., 2004; Singh et al., 2013), and frequency of teleworking (Glass & Noonan, 2016; López-Igual & Rodríguez-Modroño, 2020). We therefore consider whether children below the age of 15 are living in the household. Ethnic differences or migration background were seldom taken into account when telework was analysed, but at least one study found that ethnic minorities were under-represented in working from home (Felstead et al., 2001). We therefore control for migration background.

With regard to company characteristics, higher shares of teleworkers were found in large firms (Brenke, 2016). Although other findings suggest that firm size was not important for telework (Glass & Noonan, 2016; Peters et al., 2004), we will consider the number of employees in the estimations. We think company size might capture some degree of unobserved heterogeneity in the work environment that might be related to telework, i.e. anonymity among the employees, or pressure on the management to comply with up-to-date work standards.

### Associations with Telework at the Country Level

Access to flexible working arrangements is strongly dependent on country contexts, as the nature of flexible working is shaped within the countries’ legislation and culture (Steinmetz, 2012; Lott, 2015; Chung, 2018, 2019; Pfau-Effinger, 2010). Within Europe, there were large differences in the prevalence of telework. In 2019, the shares of telecommuters were under 10% in nearly half of the member states of the European Union, whereas in Sweden, Finland, Luxembourg and the Netherlands more than 30% of employees worked at least sometimes from home (Milasi et al., 2020). It is not surprising that in countries with larger shares of employment in knowledge- and IT-intensive services, telework is more widespread (Milasi et al., 2020). However, there were sizable differences in teleworkers’ shares across countries even within the same sector. In Sweden and the Netherlands, for instance, more than 60% of the workers in knowledge-intensive business services were teleworking, but this fraction was below 30% in Italy and even lower in Austria and Germany (Milasi et al., 2020). It is therefore important to control for country-level differences when analysing a potential gender gap in teleworking in Europe.

Furthermore, country-specific rates of female (part-time) employment or the availability of public day-care (Allen et al., 2015), or other factors, that might contribute to gender equality, may partly explain the cross-country variation in telework prevalence. It was estimated, for instance, that 22% of the variance in workers’ control over their schedules in the EU-27 (Chung, 2019) and 23% of the variance in women’s access to flexitime (Chung, 2018) was due to differences at the country level. Against this background, we will explore to what extent the individual probability of teleworking was dependent on country-level particularities, making use of multilevel regression (Snijders & Boskers, 2012).

In more gender equal countries that are characterized by equal participation of men and women in many realms of society, including the labour market, we can expect smaller gender gaps in teleworking, as vertical occupational gender segregation in such countries is relatively low (Bericat & Sánchez-Bermejo, 2019). On the other hand, comparative research suggests that gender-typed career aspirations are especially pronounced in affluent, “post-materialist” societies (Charles & Bradley, 2009; Charles, 2017). In these societies concerns about existential security are less salient in career choices, whereas cultural narratives emphasize self-fulfilment (Inglehart and Welzel 2005). Therefore, career aspirations may be built more often on (gender) stereotypes in these contexts (Charles & Thébaud, 2018). In line with this idea, horizontal occupational gender segregation was found to be stronger in more gender equal countries, i.e. in Sweden, than in more gender conservative countries (Rosenfeld & Kalleberg, 1991; Blackburn et al., 2000). Therefore, it is possible that the high shares of telework in the Nordic countries of the European Union do not reflect gender equality. As the fields of science and engineering, which are often stereotyped as ‘male’ work fields (Thébaud & Charles, 2018), offer technically advanced workplaces well suited for teleworking, a strong under-representation of women in these fields might explain part of the gender-gap in telework.

Lastly, the relative size of the public sector might be a factor for country-specific variance in the gender-gap in teleworking. Welfare states with large public sectors tend to have high female labour participation (Mandel & Semyonov, 2006). As telework was more widespread in the public compared to the private sector in the EU (Eurofound, 2007; Eurofound and International Labour Office, 2017), the gender gap in telework might be less severe in countries with a large public sector.

*We hypothesize that a significant proportion of the variance in telework is attributable to country-level characteristics (H5a), and that gender equality, occupational gender segregation, and the size of the public sector all contribute to explaining this variance (H5b).*

## Data, Variables and Method

### Data and Analytical Sample

Data are taken from the sixth European Working Conditions Survey (EWCS), which was collected in 2015 (Parent-Thirion et al., 2017). It comprised the 28 countries of the European Union plus 8 other countries, but we restricted the analysis to the EU-28. One reason for this restriction is that we made use of the Gender Equality Index from the European Institute for Gender Equality (EIGE) that is available for these countries only; it will be described below. Another reason is that the European Union aims at harmonizing labour market conditions between its member states. Therefore, the share of variance in telework attributable to structural and cultural differences between the EU member states is probably smaller than across the EU borders. If the following analysis finds variance attributable to the country level within the European Union, even larger country-level variance can be expected across other states.

The EWCS is based on random samples of workers aged 15 and older (16 and older in Bulgaria, Norway, Spain and the UK) living in private households and in employment. ‘Employment’ is defined as having done at least one hour of work for pay or profit during the preceding week, either employed or self-employed. We selected respondents who characterized their situation as being currently in work as an employee. In addition, the sample was restricted to those who made use of information or communication technology (ICT) during at least a quarter of their working time, which is considered ICT work on a regular basis (Eurofound and International Labour Office, 2017; Parent-Thirion et al., 2017). Table 1 shows that 56% of the employees pertain to this group. After data cleaning, N = 15,967 persons remained in the analytical sample.

**Table 1:** Sample definition

Criterion	N	Percent	Percent
Currently at work as an employee in EU-28	28,365	100.4	
- reported on ICT use and ISCO-08 group	28,263	100.0	
- ICT use during at least ¼ of the working time ^a^	16,056	56.8	100.0
- reported on telework	15,984		99.6
- had not more than 3 missing values on covariates ^b^	15,967		99.4

*Notes*: European Working Conditions Survey 2015.

^a^ ICT = Information and communication technology; see Table 2 for a description of included and excluded occupational groups.

^b^ Analytical N = 15,967 employees from 28 EU countries.

Table 2 gives an overview of the distribution of included and excluded employees per occupational group. Whereas the proportions of ICT workers were more than 80% in each of the major groups of managers; professionals; technicians and associated professions; and clerical support workers, the prevalence was lower in service and sales workers (44%). Much lower proportions of ICT workers were found in the major groups skilled agricultural, forestry and fishery workers; craft and related trades workers; and plant and machine operators and assemblers. In each of these major groups were sub-groups with less than 25% ICT workers; these sub-groups are highlighted in the last column of Table 2. In all the sub-groups pertaining to elementary occupations, i.e. cleaners and helpers, or street and related sales and service workers, ICT work was rare. In elementary occupations, women were over-represented. However, women were strongly under-represented among craft and related trade workers as well as among plant and machine operators and assemblers, another two large groups with few telework possibilities.

**Table 2:** ICT^a^ use during at least one quarter of employees’ working time per occupational group in the EU-28

ISCO-08 major group	No (%)	Yes (%)	N	% female of N	< 25% of workers in these sub-major groups make use of ICT according to definition
0 Armed forces occupations	26.2	73.9	132	8.2	
1 Managers	6.5	93.5	1,444	44.6	
2 Professionals	14.1	85.9	5,507	63.5	
3 Technicians and associated professionals	15.9	84.1	3,569	53.5	
4 Clerical support workers	10.9	89.1	3,003	70.2	
5 Services and sales workers	56.2	43.8	5,996	68.6	
6 Skilled agricultural, forestry and fishery workers	80.7	19.3	269	28.0	61 Market-oriented skilled agricultural workers 63 Market-oriented skilled forestry, fishery and hunting workers
7 Craft and related trades workers	75.3	24.7	3,201	15.3	71 Building and related trades workers 75 Food processing, woodworking, garment and other craft and related trades workers
8 Plant and machine operators and assemblers	75.3	24.7	2,145	20.6	82 Assemblers 83 Drivers and mobile plant operators
9 Elementary occupations	88.9	11.1	2,997	62.6	91 Cleaners and helpers 92 Agricultural, forestry and fishery labourers 93 Labourers in mining, construction, manufacturing and transport 94 Food preparation assistants 95 Street and related sales and service workers 96 Refuse workers and other elementary workers
Total (row percentages)	42.2	57.8	100.0	53.8	
Total (number of respondents)	12,207	16,056	28,263	14,958	

*Notes*: European Working Conditions Survey 2015; percentages design weighted.

^a^ ICT = Information and communication technology.

### Operationalization of Telework and Associated Characteristics

The dependent variable is telework from home at least several days a month, which meets the requirements of *regular* telework. The underlying question was: “Please take a look at these locations. In a moment, I will ask you how often you have worked in each location during the last 12 months in your main paid job / since you started your main paid job: … Your own home” with the categories “daily”, “several times a week”, “several times a month”, “less often”, and “never”. Dichotomizing working at home at the category “several days a month” ensures the inclusion of workers who usually spend one day per week working at home. Respondents who did not answer the question about working at home were excluded (less than 1% of the sample, see Table 1).

Table 3 depicts the distribution of other regular working sites for regular teleworkers and other employees. The percentages add up to more than 100% because many employees worked in additional locations to their employer’s premises and their own home. Telecommuters working from home worked more often at other locations compared to other employees not defined as telecommuters because they did not, or at most occasionally, work from home. This underscores the high flexibility of teleworkers regarding *where* they work.

**Table 3:** Working sites^a^ of regular telecommuters^b^ and other employees working with ICT^c^ in the EU-28

	Telecommuters (column percentages)	Other employees (column percentages)
At employer’s business premises	91.1	92.4
At client’s premises	32.9	16.8
In a car or other vehicle	29.1	15.0
At an outside site	20.4	12.4
In public spaces	18.3	6.9
Total (row percentages)	18.4	81.6
Total (number of respondents)	2,931	13,036

*Notes*: European Working Conditions Survey 2015; N = 15,967; percentages design weighted.

^a^ Where employees work at least several times a month.

^b^ Working from home at least several times a month.

^c^ Working with information and communication technology during at least a quarter of their working time.

Individual level variables refer to characteristics of the respondent, his or her household and work. The following characteristics might not be self-explanatory.

Migration background applies if either the respondent or at least one of his or her parents were not born in the country of residence.

The respondent’s educational level was estimated with the International Standard Classification of Education (ISCED). Preparatory analyses revealed that the lower level categories could be combined to form the category “up to lower secondary”. In the analysis, the modal category “upper secondary level” is used as reference. The other three upper levels are “post-secondary non-tertiary level”, “bachelor’s degree”, and “master’s degree and higher”.

The presence of a spouse or cohabiting partner, and of one or more children under 15 years of age are analysed separately. A small share of under 1% refused to say with whom they lived. As preparatory analyses revealed that refusing answers on household composition was related to teleworking, we decided to retain these persons in the sample and control for them in the estimations. Information about the number of children or household members is not included, as it was not additionally significant.

Commuting distance was estimated in hours and minutes per day in total spent for the one-way journey between home and the workplace; it ranges from 1 to 400 min, that is 3 h and 20 min each way. A share of 2% of the respondents reported not having any commute from home to work; these are analysed separately. An additional 1% of the respondents did not know the commuting distance or refused to answer. In order not to lose these cases, missing answers on commuting distance were imputed, and the imputed values are controlled for in the estimations.

The information on whether the respondent worked part-time comes from the respective survey question.

The occupational group of the respondent’s current job was coded on the basis of the International Standard Classification of Occupations (ISCO08). The odds of teleworking for the groups of managers, technicians, clerical support workers, and service and sales workers, are estimated against the reference group of professionals. All other occupational groups were combined to form the category “other”.

A description of the distribution of the ICT workers’ characteristics can be found in Table A1 in the supplement.

At the country level, the degree of gender equality is estimated with the Gender Equality Index (GEI), which is an indicator calculated by the European Institute for Gender Equality (EIGE, 2015). It comprehensively measures the degree of gender equality in the European Union in six core domains (work, money, knowledge, time, power and health) and two additional domains (violence against women and intersecting inequalities) (Bericat & Sánchez-Bermejo, 2019). The GEI is therefore well suited for testing the relevance of a society’s level of gender inequality with regard to telework. The indicator’s range is theoretically between 1 and 100, where 100 reflects total equality between women and men. In the sample, the GEI ranges from 50 to 83, with a mean of 65.

**Figs. 1a and 1b:**
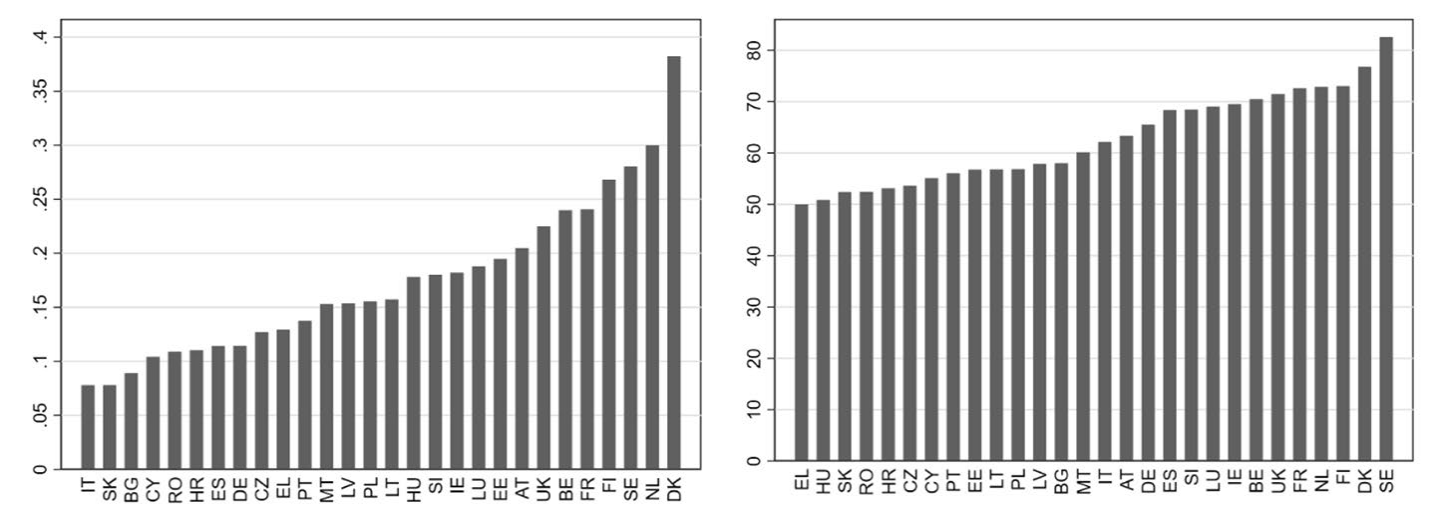
Share of telework^a^ (left) and Gender Equality Index (right) across the EU-28 *Notes*: European Working Conditions Survey 2015 (left). European Institute for Gender Equality 2015 (right). ^a^ Among N = 15,967 employees working with information and communication technology during at least a quarter of their working time.

Figure 1a and b show the distributions of the share of telework and of the gender equality index across the 28 EU countries. In the Nordic countries, the use of telework was particularly widespread compared to other EU countries, but rarely exceeded a 30% share (Fig. 1a). Telework was seldom practised in the Eastern European countries Slovakia, Bulgaria and Romania, and also seldom in Italy and Cyprus. In each of these countries, less than 15% of the ICT users worked at home on a regular basis.

Figure 1b shows the level of the Gender Equality Index (GEI) in the 28 EU countries. On the one hand, we see some consistency with the distribution of telework. Again, the Nordic countries Sweden, Denmark and Finland are at the top of the range, while some Eastern and Southern European Countries are at the bottom. For other countries, however, there is a mixed picture, resulting in a relatively low correlation between the Gender Equality Index and the percentage of teleworkers (Pearson’s r = 0.14).

As occupational gender segregation was found to be more pronounced in more gender equal countries, i.e. in Sweden, than in more gender conservative countries (Rosenfeld & Kalleberg, 1991; Blackburn et al., 2000), we additionally analyse two other factors that might play a part at the country level. As an indicator of horizontal gender segregation, the share of women compared to men in the NACE work domain “science and engineering” per country is analysed (Eurostat 2015). It ranges from 0.39 to 1.34 with a mean value of 0.75. Lastly, the impact of public sector size on the variance in telework at the country level is estimated, based on the percentage of employees working in the public sector. This measure was retrieved from the individual level data in the EWCS 2015; it ranges from 22 to 50% with a mean value of 36%.

We refrain from additionally controlling for gross domestic product (GDP), as it is highly correlated with the gender equality index (Pearson’s r = 0.73). The gender equality index is moderately correlated with the percentage of public sector employees (Pearson’s r = 0.40); other correlations among the three country-level variables are low.

### Methods of Analysis

The analysis methods comprise multivariate logistic regression with country fixed effects and multilevel regression. In a first step, country fixed effects models (Snijders & Boskers, 2012) for subsamples of men and women were used to assess possible differences in the associations of individual, household and work characteristics with telework among men and women. With this strategy, moderators of a possible gender gap in telework on the individual level can be assessed, while controlling for country particularities.

In a second step, multilevel logistic regression (Snijders & Boskers, 2012) was used to test whether a possible gender gap in telework is significant, and if so, whether it can be explained statistically. The respondents (N ≈ 16,000), who comprise the first level of estimation, were nested in one of the 28 countries of the European Union, which comprised the second level of estimation. At level one, individual and household characteristics, and characteristics related to the respondent’s work were analysed with fixed effects; at level two, country characteristics were analysed with random effects in order to allow for different levels of telework per country. This analytical strategy takes into account that individuals were exposed to similar work environments within their countries, for instance with regard to gender equality. It results in more precise estimates of individual level associations with the outcome of interest, here, telework. Additionally, applying multilevel analysis allowed us to estimate the share of variance in teleworking that was attributable to country-level influences. The observations per country ranged from 262 to 1,324. All metric independent variables were centered at the grand mean to facilitate interpretation of their effects and to reduce correlation when forming interactions (Enders, 2013). We report average marginal effects in all models to allow for comparisons of the estimates across different models (Mood, 2010).

## Findings About Gender Differences in Teleworking from Home in Europe

### What Moderates the Gender Gap in Telework?

First, the country fixed effects analysis on telework was conducted separately for men and women to explore whether individual, work-related and household characteristics show similar or different relationships with telework across gender (Table 4, models 1 and 2). The overall predicted probabilities of telework are 17.5% for women and 19.5% for men, reflecting the observed shares (cf. Table A1 in the supplement).

**Table 4:** Associations with telecommuting^a^ for male and female ICT workers,^b^ in Europe 2015

	Model 1			Model 2			Model 3		
	Women			Men			All respondents		
	AME	SE		AME	SE		AME	SE	
*Overall predict. probability*	*0.175*	*0.004*	**	*0.195*	*0.004*	**	*0.183*	*0.007*	**
Female							-0.018	0.006	**
Age	0.001	0.000	*	0.001	0.000	**	0.001	0.000	**
Migration background	-0.018	0.012		-0.030	0.013	*	-0.024	0.009	**
Educational level (ref. Upper sec.)									
Up to lower sec.	-0.023	0.016		-0.026	0.016		-0.024	0.011	*
Post-secondary non tertiary	0.040	0.010	**	0.021	0.013	+	0.032	0.008	**
Bachelor	0.083	0.012	**	0.080	0.014	**	0.079	0.009	**
Master and higher	0.166	0.014	**	0.129	0.015	**	0.147	0.011	**
Lives with partner	0.003	0.008		-0.003	0.011		0.002	0.007	
Lives with child(ren) < 15	0.025	0.008	**	0.038	0.010	**	0.031	0.006	**
Commute (10 min/day)	0.002	0.001	*	0.003	0.001	**	0.003	0.001	**
Commute: none	0.301	0.020	**	0.245	0.021	**	0.273	0.016	**
Part-time working	-0.026	0.010	**	-0.007	0.016		-0.018	0.008	*
Firm size (ref. <10 employees)									
10–245 employees	-0.015	0.012		-0.034	0.015	*	-0.022	0.009	**
250 + employees	-0.043	0.012	**	-0.045	0.015	**	-0.043	0.010	**
Private sector	-0.037	0.008	**	-0.014	0.009		-0.025	0.006	**
Occup. group (ref. Professionals)^c^									
Managers	0.018	0.018		0.003	0.019		0.010	0.013	
Technicians and associated	-0.113	0.011	**	-0.105	0.015	**	-0.112	0.009	**
Clerical support workers	-0.140	0.012	**	-0.190	0.017	**	-0.161	0.011	**
Service and sales workers	-0.144	0.013	**	-0.206	0.015	**	-0.173	0.011	**
Others	-0.164	0.020	**	-0.169	0.015	**	-0.155	0.012	**
*Country level characteristics*									
Gender Equality Index							0.004	0.001	**
Share women in science/engineer.							-0.061	0.028	*
Pct. employees in public sector							0.208	0.116	+
Number of respondents	8890			7077			15,967		
Number of countries	28			28			28		
LR/Wald χ2 (df)	1406.9	(48)	**	1226.6	(48)	**	1658.6	(25)	**
McFadden R2	0.171		**	0.176		**			
LR test: χ2 (df)							30.3	(1)	**
ICC							0.020	0.007	**

*Notes*: Data from European Working Conditions Survey; European Institute for Gender Equality; Eurostat; all 2015. AME: average marginal effects; SE: standard error. Controls for information on partner/child in household missing, and commute missing omitted. Models 1 and 2: Country fixed effects logistic regression; country variables omitted. Model 3: Multilevel logistic regression with random intercept. +p < 0.1; * p < 0.05; ** p < 0.01

^a^ At least several times a month

^b^ Employees working with information and communication technology during at least a quarter of their working time

^c^ According to ISCO-08 classification

With regard to work characteristics, some differences between men and women are revealed. In line with our expectation, part-time work was connected with a decreased probability of telework, but exclusively for women to a significant extent. Additionally, working in the private sector was more strongly associated with a decreased probability of teleworking for women than for men.

The longer the commute between home and the workplace, the higher the probability of telework, whereas those who did not have any commute most often worked at home. Commuting distance was more relevant for telework among men, whereas having no commute was more relevant for women, but the differences were not pronounced.

In line with previous research (Singh et al., 2013; Elldér, 2019; López-Igual & Rodríguez-Modroño, 2020; Brenke, 2014, 2016), we find that telework was associated with working in certain occupational groups, namely managers and professionals. Technicians had a significantly lower probability of teleworking compared to professionals; for all other groups the probabilities were even lower. Additionally, holding tertiary educational degrees was associated with higher probabilities of teleworking, also corroborating previous findings (Brenke, 2016; López-Igual & Rodríguez-Modroño, 2020; Elldér, 2019; Singh et al., 2013). The differences between men and women were small.

The presence of children in the household was positively associated with teleworking from home, as expected (Elldér, 2019; Glass & Noonan, 2016; López-Igual & Rodríguez-Modroño, 2020), but more strongly for men. However, the gender difference was not statistically significant.

### Explaining the Gender Gap in Telework Within a Multilevel Framework

In Table 4, model 3, mixed effects logistic regression (Snijders & Boskers, 2012) is applied to analyse the amount of variance in the dependent variable, regular telework from home, attributable to the country level, and to test the associations between telework and gender as well as other characteristics. The first row of estimates in Table 4 shows the overall predicted probability of telework, which is 18.3% in the total sample of model 3. In the following, we will interpret the estimates in model 3 dependent on this overall probability.

For women, a 10% (1.8/18.3) lower probability of telework is estimated compared to men. This gender gap is considerably lower than previously suggested (between 25% and 32%; López-Igual & Rodríguez-Modroño 2020), which underscores the notion that our approach is more conservative. Nevertheless, the gender gap is significant. We additionally tested whether allowing the slope of gender to vary across the countries would improve the estimation (not displayed). The variance term was small and barely statistically significant. Additionally, the Wald χ^2^ indicated a poorer fit. We conclude from these findings that there are no significant differences between the countries under study in the strength of the association between gender and telework. These findings *support hypothesis 1*, saying that female employees in Europe had a significantly lower probability of teleworking compared to men before the Covid-19 pandemic, also among ICT employees. Moreover, this finding holds net of other individual and household characteristics, as well as net of occupational and employer characteristics.

Part-time work was generally negatively associated with telework, *corroborating hypothesis 2a*. Part-time working decreased the probability of teleworking by 10% on average (1.8/18.3), net of other influences.

With regard to working in the private versus public sector, the findings lend *support to hypothesis 3a*: Working in the private sector was associated with a 14% (2.5/18.3) decreased probability of teleworking.

Commuting time was associated with an increased probability of telework to a moderate but significant extent. For every 10 min interval of commuting time, the probability of telework is estimated to have increased by 2% (0.3/18.3), which lends *support to hypothesis 4a*. This finding is remarkable, as it was obtained after controlling for having no commute at all, which is – not surprisingly – the single most important predictor, and after controlling for other relevant influences: educational level (Huber, 2012; Melzer & Hinz, 2019) and occupational position (Romaní et al., 2003; Eliasson et al., 2003). *Hypothesis 4b*, stating that avoiding lengthy commutes is more strongly associated with telework among women compared to men, *was not supported*.

Other individual level findings are as expected. Higher incidences of telework were associated with higher levels of education; higher occupational position; and lower numbers of employees in a company. Having children under 15 in the household was associated with an increased likelihood of teleworking, having a migration background with a decreased one.

Next, we report the findings regarding the question whether the moderators revealed above were able to “explain” the gender gap in telework. Interaction effects in logit models are difficult to interpret without visualization (Mize, 2019); therefore, further analyses are presented graphically. We chose the GEI for displaying the range of the effects across the countries (for the GEI level of specific countries, see Fig. 1b).

**Figs. 2a and 2b:**
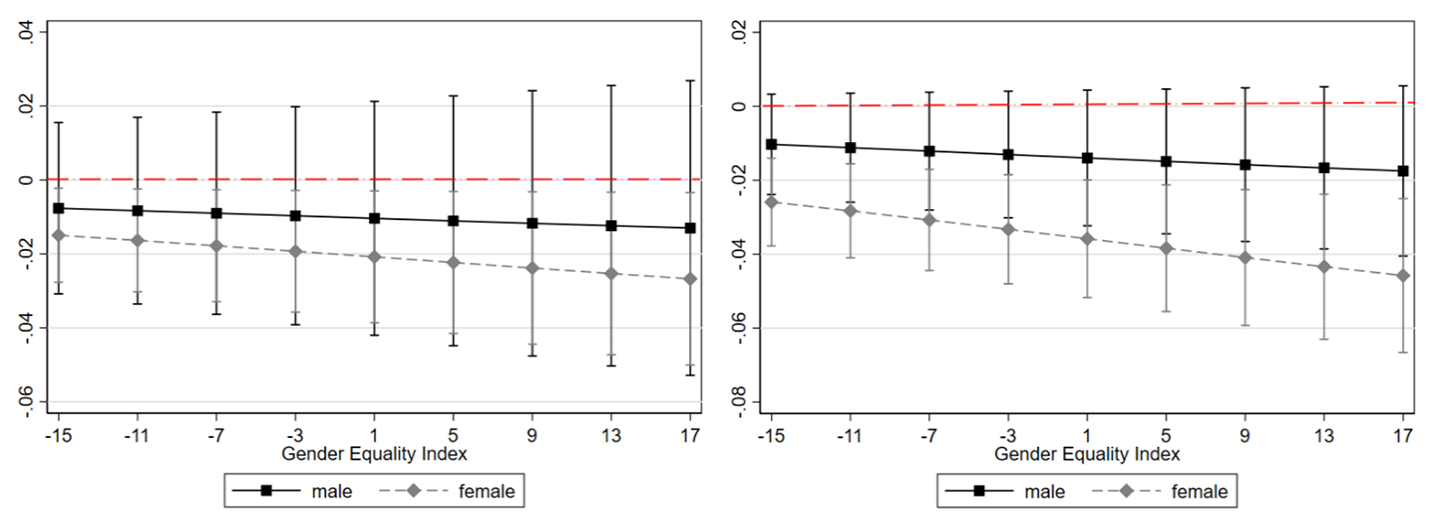
Average marginal effects of working part-time (left) and in the private sector (right) on telework for men and women in relation to Gender Equality Index per country, EU-28 *Notes*: Data from European Working Conditions Survey; European Institute for Gender Equality; Eurostat; all 2015. Estimates on basis of model 3 in Table 4 including respective interaction terms. N = 15,967 employees working with ICT.

The interaction effect of part-time work with gender is plotted in Fig. 2a, with reference to the gender equality index across the countries. The plot reveals that part-time work was associated with a lower probability of teleworking to a significant extent exclusively for women, but not for men. This finding is in line with *hypothesis 2b*. The interaction effect of working in the private sector with gender is plotted in Fig. 2b. Again, exclusively for women, working in the private sector was associated with significantly lower probabilities of telework, *corroborating hypothesis 3b*. These findings suggest that the negative outcomes of working part-time and in the private sector *for woman* largely “explain” the estimated gender gap in telework.

**Figs. 3a and 3b:**
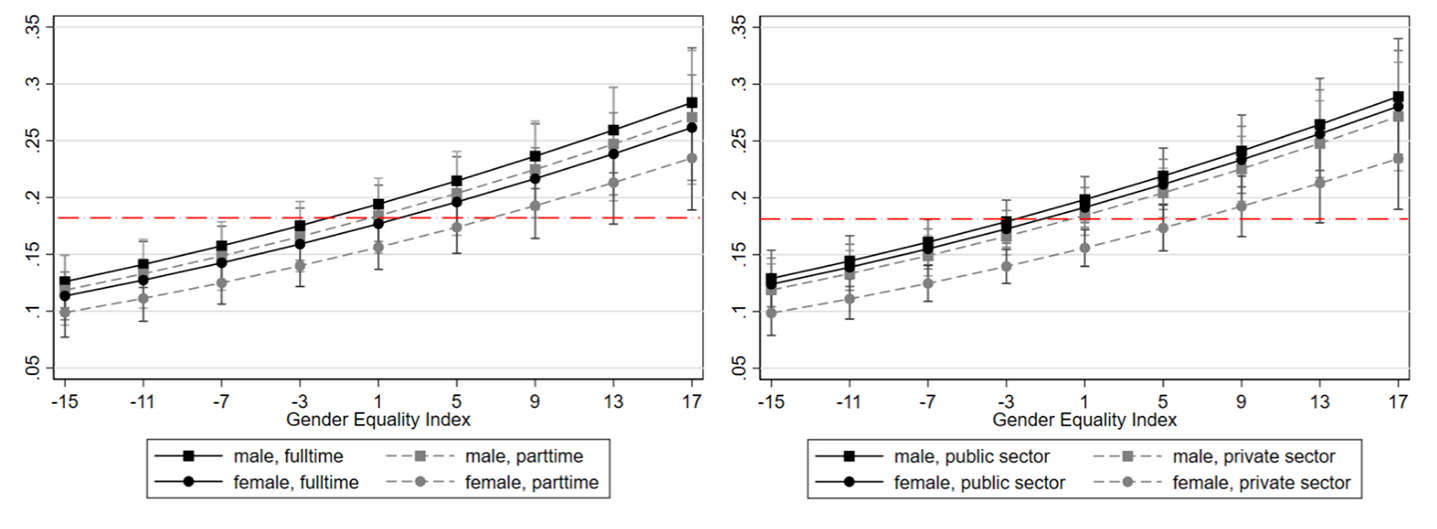
Marginal predicted means of working part-time (left) and in the private sector (right) on telework for men and women in relation to Gender Equality Index per country, EU-28 *Notes*: Data from European Working Conditions Survey; European Institute for Gender Equality; Eurostat; all 2015. Estimates on basis of model 3 in Table 4 including respective interaction terms. N = 15,967 employees working with ICT.

In the following, we depict the marginal predicted means of teleworking for women and men in the countries under study, dependent on working part-time and in the private sector. Both Fig. 3a and b show a similar picture. Telework is more likely in countries ranking high on the gender equality index compared to countries ranking low on it. In countries ranking low on the gender equality index, men and women, regardless of working full-time or part-time and in the private or the public sector, have lower probabilities for teleworking than on average in the European Union. Starting at medium levels of gender equality, the disadvantage of female part-timers and women in the private sector becomes evident. For men, neither working part-time nor working in the private sector is decisive for telework.

According to the empty Model 0 (not displayed), 6.5% of the variance in telework is attributable to the country level (ICC = 0.065; Std. Err. = 0.017). This share is rather low but significant, which *corroborates hypothesis 5a*. However, the small intraclass correlation coefficient (ICC) indicates that the main associations with telework were found at the individual and household level, whereas country particularities explain only a small part.

**Figs. 4a and 4b:**
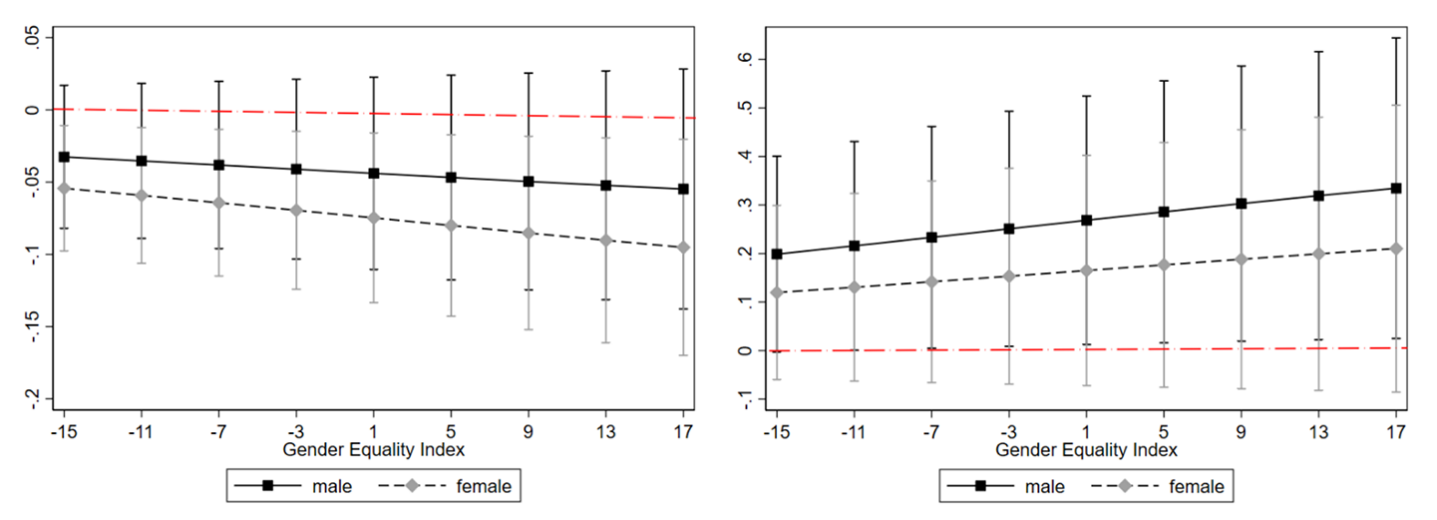
Average marginal effects of share of women in science and engineering (left) and percent employees in the public sector (right) on telework for men and women in relation to Gender Equality Index per country, EU-28 *Notes*: Data from European Working Conditions Survey; European Institute for Gender Equality; Eurostat; all 2015. Estimates on basis of model 3 in Table 4 including respective interaction terms. N = 15,967 employees working with ICT.

At the country level, gender equality, the share of women in STEM fields, and the extent of the public sector explain the variation in telework across the European countries largely, *corroborating hypothesis 5b*. The higher a country ranks on the Gender Equality Index (GEI), the higher the probability of telework was for its citizens; the stronger women were represented in science and engineering the lower the probability of telework was; and the larger the public sector the higher the probability of telework was. Figure 4a shows that higher shares of women in science and engineering were related to higher teleworking rates for both men and women, but more strongly for men. Figure 4b shows that the larger the public sector was, the less likely telework was for both men and women, but only for women to a significant extent. Effect sizes increased the higher a country ranked on the gender equality index with regard to both these relationships.

The fit of the models in Table 4 is good: The McFadden R^2^ in the single-level, gender-specific models is 0.17 to 0.18. In the multilevel models, the intraclass correlation is small (6.5% in the empty model), but the LR-chi^2^ test indicates a significant improvement in model fit when the level of telework is allowed to vary randomly across the countries. The inclusion of country information adds significantly to the explanation of telework, and it reduces the intraclass correlation strongly. Only 2% of the variance in regular telework from home at the country level remains unexplained in the full model.

Sensitivity analyses show that our results are stable even when a higher proportion of ICT use during employees’ working hours or a higher frequency of telecommuting is applied (see Table A2 and Figures A1 and A2 in the supplement). Moreover, the main results remain when self-employed workers are included and even when those who do not commute are excluded. The gender gap in telework across model specification ranges from 9 to 16% (Table A3).

## Discussion and Conclusion

The goal of this paper was to explore whether there was a significant gender gap in telework before the Covid-19 pandemic in Europe, which is a question of distribution at the macro-level, and to analyse its possible constituents at the micro-level of individual employees embedded in socio-economic environments. We restricted the analytical sample to ICT employees, because only they had the possibility to telework outside their employer’s premises. The main part of the analysis therefore was based on a sample of *potential* teleworkers, male or female, so that a large part of *horizontal* gender segregation already was accounted for: the fact that men and women were concentrated in certain fields of work and occupations. Descriptive material was presented to illustrate to which extent employees from different occupational groups were included in or excluded from the analytical sample.

Telework was defined as working from home several times a month or more often, which included the common arrangement of one-day telework per week, but excluded instances of occasional work from home, e.g. because the child was sick. Our operationalization of home-based telework followed the definition provided by Eurofound and International Labour Office (2017) for measuring regular work with ICT from home with EWCS data. *Regular* telework is what we have in mind when discussing emerging trends in the aftermath of the pandemic, i.e. the substitution of office space through teleworking from home.

The results show that before the pandemic, there was a significant gender gap in teleworking of 10% in the EU-28, i.e. women were under-represented in telework compared to men. This gap is smaller than earlier estimates (López-Igual & Rodríguez-Modroño, 2020) because it was obtained while controlling for large parts of horizontal occupational gender segregation. Moreover, this finding holds net of other socio-demographic, household, and work-related characteristics. Therefore, we consider this gender gap in telework substantial.

Working part-time and working in the private sector were both found to moderate the under-representation of women among teleworkers, and together they largely explain the gender gap in telework statistically. Based on the existing body of research we are inclined to speculate about which “mechanisms” may lie behind these findings. First, the findings are possibly best explained by putting them in historical perspective (Blackburn et al., 2002; Thébaud & Charles, 2018). The fields of work where telework was established and spread first, computer science and telecommunications (Kraut, 1994; Kizza, 2017), were highly gender segregated fields with a strong male dominance in the Western world (Charles & Bradley, 2009; Cheng et al., 2019). The ideas of the “brilliant” scientist, and the maths or programming “nerd”, who are exclusively focused on their scientific or technological endeavours, do not only deter young women from embarking on a career in STEM fields (Thébaud & Charles, 2018), but are also incompatible with working part-time. Historically, telework was a male domain, whereas part-time work was a domain of working mothers (Drobnic, 2000). The future spread of telework in Europe might also considerably depend on the development of telework availability in public sector work, where part-time working women are over-represented (Mandel & Semyonov, 2006).

Another notable finding of this study is that commuting distance also matters for teleworking in Europe. Already before the Covid-19 pandemic, the share of exclusively home-based ICT employees was significant, but at a low level (2% of potential teleworkers). If employees commuted, then the likelihood of telework increased with commuting distance for both men and women. This finding underscores the relevance of telework for questions of settlement development, transportation planning, and therefore also for the development of urban sprawl and CO_2_ emissions.

Country particularities were found to have accounted for a small but significant share of the variance in telework across the EU countries; it was estimated at 6.5%. In light of EU integration policies that strive for both the alignment of technological development and harmonized labour markets, the share of variance in telework applicable to the country level was probably larger across European borders than within the European Union. Employees living in more gender-equitable countries were found to have had higher probabilities of telework compared to those in less gender-equitable ones, measured with the Gender Equality Index (GEI). However, our analysis suggests that in contexts with high gender equity, particularly men teleworked more often compared to women. As men’s flexible working was found to support their spouses part-time working (Buchler & Lutz, 2021), such a combination with telework might be particularly widespread in countries with high gender equity.

Potential shortcomings of this study lie in the cross-sectional character of the data that allows the assessment of associations but not of causal effects. The analysis was based on a relatively low number of 28 countries, which is close to the minimum number required for multilevel analysis to yield reliable results (Bryan & Jenkins, 2016). Furthermore, the data did not allow for differentiating between workers’ demand for and employers granting permission of telework. Therefore, we cannot make inferences about whether gender discrimination might play a part in explaining the gender gap in teleworking found here.

Nevertheless, this study explored associations between gender and telework more comprehensively than previous ones. To the best of our knowledge, this is the first study to analyse telework with a multilevel design. It found a moderate gender gap in telework and it localized a small but significant part of the explanation of telework incidence at the country level. We consider these findings a good starting point for further analyses of the determinants and outcomes of telework after the Covid-19 pandemic.

## Electronic Supplementary Material

Below is the link to the electronic supplementary material.

Supplementary Material 1

## Data Availability

The datasets analyzed during the current study are available at the European Foundation for the Improvement of Living and Working Conditions: https://www.eurofound.europa.eu/surveys/about-eurofound-surveys/data-availability#datasets; at the European Institute for Gender Equality: https://eige.europa.eu/gender-equality-index/2015; and at Eurostat: https://ec.europa.eu/eurostat/data/database.

## Code Availability

Code files will be made available on request.
