# Supplementary material for: Exploring the Gender Gap in Teleworking from Home. The Roles of Worker’s Characteristics, Occupational Positions and Gender Equality in Europe
Source: Soc Indic Res. 2023 May 24:1–22. Online ahead of print. doi: 10.1007/s11205-023-03133-6 (PMC10206342; doi:10.1007/s11205-023-03133-6)
Supplement: Supplementary file 1 — Supplementary Material 1 [file 11205_2023_3133_MOESM1_ESM.pdf]

Exploring the gender gap in teleworking from home. The roles of worker's characteristics, occupational positions and gender equality in Europe

Supplement

Table A1: Characteristics of ICT workers in the EU-28[a]

	Women	Men	All respondents			
	Mean	Mean	Mean	Std. Dev.	Min.	Max.
Dependent: telecommuting[b]	0.18	0.20	0.18	0.39	0	1
Female	1.00	0.00	0.56	0.50	0	1
Age	42	42	42	11	15	80
Migration background	0.13	0.13	0.13	0.34	0	1
Educational level						
Up to lower secondary	0.06	0.09	0.07	0.25	0	1
Upper secondary	0.33	0.34	0.33	0.00	0	1
Post-secondary non-tertiary	0.24	0.21	0.23	0.42	0	1
Bachelor	0.20	0.18	0.19	0.39	0	1
Master and higher	0.17	0.18	0.18	0.38	0	1
Lives with partner	0.70	0.72	0.71	0.45	0	1
Lives with child(ren) <15 years	0.34	0.32	0.33	0.47	0	1
Lives with: information miss.	0.00	0.00	0.00	0.07	0	1
Commute (total), minutes/day	43	48	45	36	1	400
Commute: none	0.02	0.03	0.02	0.15	0	1
Commute: information miss.	0.01	0.01	0.01	0.09	0	1
Part-time work	0.24	0.08	0.17	0.38	0	1
Firm size						
<10 employees	0.18	0.13	0.16	0.00	0	1
10-245 employees	0.44	0.41	0.43	0.49	0	1
250+ employees	0.38	0.46	0.41	0.49	0	1
Private sector	0.55	0.66	0.60	0.49	0	1
Occupational group[c]						
Managers	0.06	0.10	0.08	0.27	0	1
Professionals	0.33	0.26	0.30	0.00	0	1
Technicians	0.18	0.20	0.18	0.39	0	1
Clerical support workers	0.22	0.10	0.17	0.37	0	1
Service and sales workers	0.18	0.13	0.16	0.37	0	1
Others	0.04	0.20	0.11	0.31	0	1
N of employees	8,890	7,077	15,967			

Notes: Data from European Working Conditions Survey 2015. Estimates design-weighted.
[a] Employees working with information and communication technology during at least a quarter of their working time.
[b] At least several times a month.
[c] According to ISCO 08 classification.

Table A2: Sensitivity analyses.
Associations with telecommuting[a] for male and female ICT workers[b], in Europe 2015

	Model 4			Model 5		
	AME	SE		AME	SE	
Overall predict. probability	*0.191*	*0.008*	**	*0.112*	*0.006*	**
Female	-0.030	0.007	**	-0.010	0.005	*
Age	0.001	0.000	**	0.001	0.000	**
Migration background	-0.013	0.010		-0.018	0.007	*
Educational level (ref. Upper sec.)						
Up to lower sec.	-0.019	0.015		-0.014	0.009	
Post-secondary non tertiary	0.029	0.010	**	0.023	0.007	**
Bachelor	0.076	0.011	**	0.056	0.008	**
Master and higher	0.137	0.012	**	0.083	0.009	**
Lives with partner	-0.002	0.008		-0.004	0.005	
Lives with child(ren)<15	0.038	0.008	**	0.021	0.005	**
Commute (10 minutes/day)	0.004	0.001	**	0.001	0.001	
Commute: none	0.304	0.020	**	0.221	0.013	**
Part-time working	-0.017	0.010		-0.011	0.007	
Firm size (ref. <10 employees)						
10-245 employees	-0.023	0.012	*	-0.004	0.008	
250+ employees	-0.033	0.012	**	-0.037	0.008	**
Private sector	-0.022	0.007	**	-0.033	0.005	**
Occup. group (ref. Professionals)[c]						
Managers	0.012	0.015		-0.012	0.010	
Technicians and associated	-0.102	0.011	**	-0.087	0.008	**
Clerical support workers	-0.156	0.012	**	-0.116	0.009	**
Service and sales workers	-0.175	0.014	**	-0.109	0.009	**
Others	-0.143	0.016	**	-0.100	0.010	**
Country level characteristics						
Gender Equality Index	0.005	0.001	**	0.003	0.001	**
Share women in science/engineering	-0.036	0.033		-0.038	0.023	+
Pct. employees in public sector	0.290	0.134	*	0.181	0.093	+
Number of respondents	10,957			15,967		
Number of countries	28			28		
Wald $\chi 2$ (df)	1084.4	(25)	**	1264.7	(25)	**
LR test: $\chi 2$ (df)	48.7	(1)	**	47.0	(1)	**
ICC	0.023		**	0.025		**

Notes: Data from European Working Conditions Survey; European Institute for Gender Equality; Eurostat; all 2015.
Multilevel logistic regression with random intercept. AME: average marginal effect; SE: standard error.
Controls for information on partner/child in household missing, and commute missing omitted.
[a] Model 4: At least several times a month; Model 5: At least several times a week.
[b] Employees working with information and communication technology during at least *three quarters* (Model 4) / *one quarter* (Model 5) of their working time.
[c] According to ISCO 08 classification.
+ p<0.1; * p<0.05; ** p<0.01.

Figures A1a and A1b: Average marginal effects of working part-time (left) and in the private sector (right) on telework for men and women among employees working with ICT during at least *three quarters* of their working time in relation to Gender Equality Index per country, EU-28

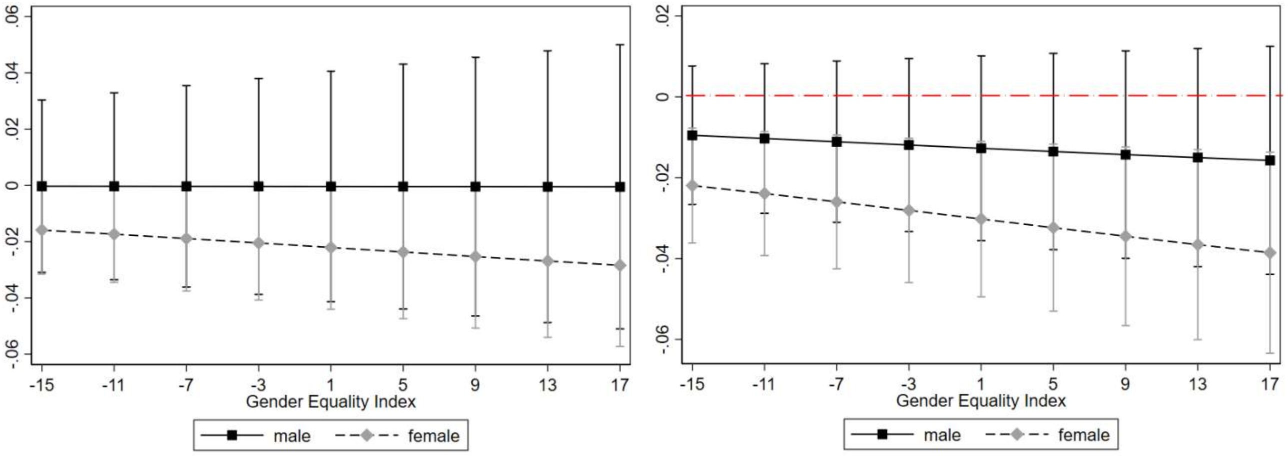

Notes: Data from European Working Conditions Survey; European Institute for Gender Equality; Eurostat; all 2015.
Estimates on basis of model 4 in Table A2 including respective interaction terms.
N=10,957 employees.

Figures A2a and A2b: Average marginal effects of working part-time (left) and in the private sector (right) on teleworking at least *several times a week* for men and women in relation to Gender Equality Index per country, EU-28

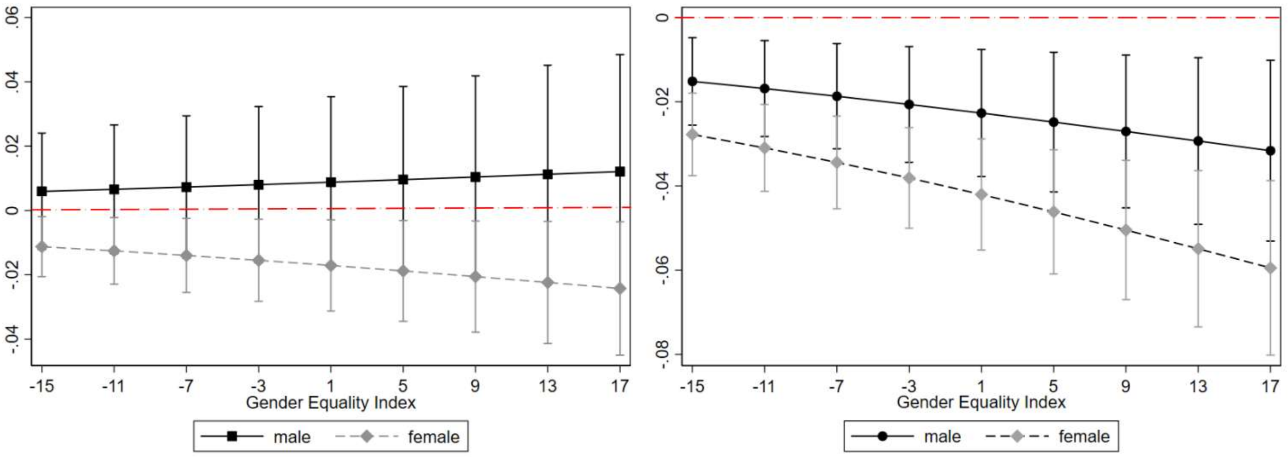

Notes: Data from European Working Conditions Survey; European Institute for Gender Equality; Eurostat; all 2015.
Estimates on basis of model 5 in Table A2 including respective interaction terms.
N=15,967 employees working with ICT during at least a quarter of their working time.

Table A3: Sensitivity analyses.
Estimated gender gaps[a] in telecommuting for ICT workers, in Europe 2015

Model 3	Model 4	Model 5	Model 6	Model 7
−10%	−16%	−9%	−11%	−11%

Model 3 (see Table 4, baseline): ICT use during at least one quarter of the working time; telework at least several times a month; controls included
Model 4 (see Table A2): baseline, but ICT use during at least *three quarters* of the working time
Model 5 (see Table A2): baseline, but telework at *least several times a week*
Model 6 (not displayed): baseline, but *self-employed included*
Model 7 (not displayed): baseline, but respondents having *no commute excluded*
[a] Average marginal effect of female / overall predicted probability of telework.
